# Laplace-guided fusion network for camouflage object detection

**DOI:** 10.3389/frai.2025.1732820

**Published:** 2026-01-14

**Authors:** Jiangxiao Zhang, Feng Gao, Shengmei He, Bin Zhang

**Affiliations:** Xingtai University, Xingtai, HeBei, China

**Keywords:** camouflage object detection, feature fusion, frequency domain, laplace-transformed, multi-scale fusion

## Abstract

Camouflaged object detection (COD) aims to identify objects that are visually indistinguishable from their surrounding background, making it challenging to precisely distinguish the boundaries between objects and backgrounds in camouflaged environments. In recent years, numerous studies have leveraged frequency-domain methods to aid in camouflage target detection by utilizing frequency-domain information. However, current methods based on the frequency domain cannot effectively capture the boundary information between disguised objects and the background. To address this limitation, we propose a Laplace transform-guided camouflage object detection network called the Self-Correlation Cross Relation Network (SeCoCR). In this framework, the Laplace-transformed camouflage target is treated as high-frequency information, while the original image serves as low-frequency information. These are then separately input into our proposed Self-Relation Attention module to extract both local and global features. Within the Self-Relation Attention module, key semantic information is retained in the low-frequency data, and crucial boundary information is preserved in the high-frequency data. Furthermore, we design a multi-scale attention mechanism for low- and high-frequency information, Low-High Mix Fusion, to effectively integrate essential information from both frequencies for camouflage object detection. Comprehensive experiments on three COD benchmark datasets demonstrate that our approach significantly surpasses existing state-of-the-art frequency-domain-assisted methods.

## 1 Introduction

In the natural world, animals, plants, and insects often employ camouflage strategies to avoid predation by either utilizing environmental features or modifying their appearance and coloration to achieve seamless integration with their surroundings (Sengar and Mukhopadhyay, 2017d, 2020b, 2017a,b, 2020a, 2017c). COD focuses on identifying such visually concealed targets within complex and deceptive scenes (Fan et al., 2020a). The core challenge of COD lies in the high visual similarity between the object and its background, which significantly increases task complexity. COD has demonstrated wide applicability across various domains, including military surveillance, medical diagnostics, and agricultural monitoring. For example, it is relevant in detecting camouflaged soldiers or equipment in military contexts, identifying and segmenting polyps in endoscopic images for medical analysis (Fan et al., 2020b), and monitoring crop growth stages in precision agriculture (Zheng et al., 2018). In these scenarios, accurate detection of camouflaged targets can be vital for operational success or early-stage diagnosis.

Owing to its broad range of practical applications, COD has garnered increasing attention from the research community and has witnessed notable advancements in recent years (Zhou et al., 2024; Sun et al., 2022; Liu et al., 2024). However, COD remains a highly challenging task due to two primary factors. The first is camouflage deception, where the object shares similar colors and textures with the surrounding background, making it difficult to achieve even coarse localization. The second is the edge perception challenge, caused by extremely ambiguous object boundaries, which significantly hinders accurate segmentation even after approximate localization is obtained. To tackle these challenges, most existing approaches focus on enhancing boundary awareness or introducing additional information to improve the detection performance and robustness of COD models. Moreover, methods that combine multi-level features from different semantic depths are often used to balance spatial detail and contextual semantics. Despite this, such fusion strategies are still limited by the intrinsic ambiguity of camouflaged targets.

Among CNN-based architectures, most existing methods improve COD by employing dual-branch structures and attention mechanisms to enhance the perception of object boundaries. While these approaches strengthen edge awareness and thus improve detection accuracy, they often overlook the underlying structural correlations between camouflaged objects and their surrounding backgrounds. Capturing these intrinsic relationships can guide the model to more effectively distinguish and segment camouflaged regions. This observation suggests that not only emphasize salient object features, but also model the mutual dependency between object and background in a more principled manner.

Recent developments in computer vision research have increasingly recognized the valuable role that frequency-domain features play in augmenting boundary recognition performance within COD systems (He et al., 2023; Le et al., 2025; Zhong et al., 2022). For example, FEDER (He et al., 2023) and FDNet (Zhong et al., 2022) leverage wavelet transforms to fuse high- and low-frequency components, thereby improving detection performance. However, wavelet-based methods present limitations: the low-frequency sub-band typically requires further decomposition to reveal semantic content, and the high-frequency sub-bands (LH, HL, HH) may carry noise or redundant details. CamoFA adopts a Fourier transform-based strategy to adaptively integrate low-frequency components from reference images with high-frequency details of the input image. While this method enhances detection capability, Fourier transforms inherently lose spatial locality, making it challenging to accurately localize object edges and often introducing redundant information.

To overcome these shortcomings, we explore the potential of the Laplace transform, which excels at highlighting regions with sharp intensity changes. By emphasizing high-frequency variations, the Laplace transform helps the network focus on fine-grained boundary features, which are critical for detecting camouflaged objects. Based on this observation, we propose a Laplace-guided framework, termed Self-Correlation Cross Relation Network (SeCoCR). SeCoCR is a dual-branch architecture: the first branch extracts global features from the original image using a Vision Transformer (ViT), while the second branch extracts local features from the Laplace-transformed image using a Convolutional Neural Network (CNN). Two key modules are introduced within this framework: Self-Relation Attention (SRA) and Low-High Mix Fusion. At each feature extraction stage, the SRA module is used to compute self-correlation representations for both global and local features, enhancing the contextual expressiveness of each. These enriched features are then fused through the Low-High Mix Fusion module, enabling effective integration of local detail and global context. This design not only strengthens the boundary sensitivity of the model but also improves its resilience to background noise, thereby achieving a better balance between precision and generalization.

In summary, our main contributions are as follows:

- We propose a new COD framework, termed Self-Correlation Cross Relation Network (SeCoCR). This framework introduces a new perspective by explicitly incorporating Laplace-based frequency-domain information into the detection process, enabling more accurate localization and segmentation of camouflaged targets that exhibit minimal contrast with the background.
- We design two key components within the SeCoCR architecture to facilitate effective feature learning. First, the Self-Relation Attention (SRA) module captures and reinforces intra-branch contextual dependencies, allowing the model to better preserve subtle spatial information within each stage. Second, the Low-High Mix Fusion (LHMF) module performs hierarchical integration of global semantics and fine-grained local details, yielding a unified representation that significantly boosts the discriminative power of the model.
- Extensive experiments conducted on three challenging COD benchmarks demonstrate that SeCoCR outperforms 19 state-of-the-art COD methods, showcasing its effectiveness and generalizability.

## 2 Related works

### 2.1 Camouflage object detection

COD aims to identify and segment objects that are deliberately concealed or naturally blend into their surroundings. Over the years, researchers have explored various approaches, ranging from early hand-engineered techniques to modern deep learning-based frameworks that leverage large-scale datasets and sophisticated model architectures. The advent of convolutional neural networks (CNNs) has led to a paradigm shift in COD. One of the pioneering works in this domain is SINet (Fan et al., 2020a), which introduced a two-stage architecture inspired by predator hunting behaviors. The model was designed to first search for camouflaged targets and then refine their segmentation. ZoomNet (Pang et al., 2022) and related methods simulate the human eye's ability to dynamically zoom in and out to identify potential targets at varying resolutions. These multi-scale analysis techniques exploit spatial context at both coarse and fine levels, improving the ability to detect small or faint objects that may be missed when viewed only at a single scale. PFNet Yang et al. (2021), another bio-inspired model, incorporates a focus mechanism that mimics human attention shifts. The network learns to prioritize regions with a higher likelihood of containing camouflaged objects and iteratively refines its attention maps, leading to more accurate localization. C2FNet (Sun et al., 2021), for example, emphasized the integration of contextual cues. By leveraging a coarse-to-fine strategy, it progressively refines object boundaries and improves detection precision through enhanced feature fusion. Similarly, methods such as TINet (Zhu et al., 2021), DGNet (Ji et al., 2023), and CINet (Li et al., 2023) explicitly introduced texture-aware modules, recognizing that texture dissimilarities can play a decisive role in separating camouflaged objects from their environment. More recent contributions push the boundary of COD by integrating domain-specific cues and innovative learning paradigms. EANet (Liu et al., 2024) significantly improves the recognition accuracy in hard-boundary regions by selectively focusing on key boundary features while suppressing confusing texture interference. By integrating multi-scale feature fusion and an iterative refinement strategy, it progressively generates high-resolution segmentation masks. DINet (Zhou et al., 2024) adopts a dual-branch decoder architecture to separately learn the core regions and edge details of the target. It introduces an interactive feature fusion module to dynamically integrate these two types of features and incorporates a global context unit to enhance the localization capability of the main object features. DAD (Li et al., 2025) proposes a unified difference-aware decoder that mimics the two-stage processing of the human visual system to effectively enhance foreground-background contrast in complex scenes.

### 2.2 Camouflage object detection in Frequency Domain

Several recent efforts investigate frequency domain information to uncover fine-grained boundaries and subtle texture differences. For instance, FEDER (He et al., 2023) enhances the performance of COD by leveraging a learnable wavelet-based decomposition mechanism that separates features into multiple frequency bands and selectively emphasizes the most informative ones. Additionally, an ODE-inspired edge reconstruction module is introduced to refine object boundaries, thereby improving localization precision. The Frequency Enhancement Module (FEM) (Zhong et al., 2022) employs the Discrete Cosine Transform (DCT) to extract informative frequency-domain features and applies a learnable enhancement process to emphasize meaningful patterns. To effectively integrate spatial and frequency information, a Feature Alignment (FA) mechanism is designed to align and fuse RGB and frequency-domain features. Furthermore, a High-Order Relation (HOR) module is proposed to model subtle variations between features, facilitating more accurate localization of camouflaged objects in complex scenes. CamoFA (Le et al., 2025) introduces a learnable frequency-domain enhancement strategy that combines the strengths of the Fourier Transform and Conditional Generative Adversarial Networks (CGANs). This approach adaptively integrates the low-frequency components of reference images with the high-frequency details of input images, thereby improving the model's ability for COD.

Although several existing frequency-domain-based methods have achieved notable progress in COD, our proposed SeCoCR framework introduces a fundamentally different and more effective design in several key aspects. First, we utilize the Laplace transform to enhance high-frequency components that are strongly correlated with object boundaries. This enables our model to directly highlight the contours of camouflaged objects while simultaneously suppressing redundant background textures, which is particularly beneficial in challenging low-contrast scenarios. In contrast, CamoFA (Le et al., 2025) leverages a conditional generative adversarial network (CGAN) to perform frequency-domain fusion. However, CGAN-based training is inherently unstable and sensitive to hyperparameter tuning, often leading to suboptimal convergence and inconsistent performance. FDNet (Zhong et al., 2022), on the other hand, applies a static feature alignment strategy in the frequency domain. While effective to some extent, it lacks the flexibility to capture more complex and spatially varying boundary structures present in highly camouflaged scenes. In contrast to these approaches, our SeCoCR framework employs a multi-level Low-High Frequency Mix Fusion (LHMF) strategy that enables deterministic, spectrum-guided cross-attention. This mechanism adaptively integrates complementary information from both low-frequency (global semantic context) and high-frequency (fine-grained boundary details) components across different feature hierarchies. As a result, SeCoCR can better preserve structural integrity and improve target-background separability, leading to more accurate and robust detection performance.

## 3 Methodology

Accurately locating camouflaged object regions is a key challenge in computer vision, with the main difficulty stemming from the high similarity between foreground targets and background environments in terms of texture, color, and semantic features. To address this challenge, this paper proposes SeCoCR, a dual-branch collaborative reasoning network specifically designed for COD. The core innovation of this network lies in its multi-modal feature collaborative enhancement mechanism. At each processing stage, SeCoCR extracts deep semantic features from the original image and high-frequency boundary features enhanced by the Laplacian transform through parallel branches, respectively. It also designs a fusion module for high- and low-frequency information to achieve hierarchical complementary fusion of semantic information and boundary cues, where semantic features provide regional consistency constraints, while boundary features focus on local detail enhancement. Finally, the feature reconstruction loss forces the network to sharpen target contours while preserving semantic integrity.

### 3.1 Overall architecture

**Figure 1:**
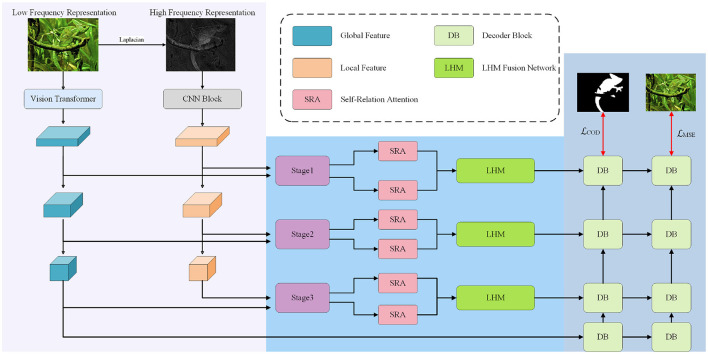
The overall architecture.

The overall framework of the proposed method is illustrated in Figure 1 and consists of two main modules across three progressive parts: Part 1: The input image undergoes a Laplacian transformation to emphasize local boundary information. This preprocessing step accentuates high-frequency components critical for identifying camouflaged object boundaries against complex backgrounds. The transformed image is then processed by a convolutional neural network to extract local features. Meanwhile, the original image is fed into a Transformer-based encoder to capture global contextual features. Part 2: For each stage in the hierarchical feature extraction process, the extracted features are refined and fused. Specifically, both local and global features are passed through the proposed Self-Relation Attention (SRA) module to emphasize informative representations and suppress noise. The SRA module adaptively recalibrates feature responses to prioritize salient regions while attenuating irrelevant activations. The enhanced features are then fused via the Low-High Mix Fusion (LHM) module, which integrates high-frequency details from the Laplacian branch with low-frequency semantics from the original image branch. This dual-branch fusion strategy ensures complementary information exchange between structural details and semantic context. Part 3: In this stage, the multi-scale features from each level are fed into a decoder to progressively reconstruct the camouflaged object mask. The fusion of different resolutions and levels enables the model to maintain fine boundary details while preserving global structure. Losses are computed across multiple scales to ensure robust supervision during training. The hierarchical supervision mitigates gradient vanishing and enhances feature discriminability.

In the Laplace-guided local branch, we adopt a convolutional neural network (CNN) to extract multi-level structural cues from the Laplacian-transformed images. At each stage, the same lightweight convolutional block is applied to capture fine edge details and texture variations around camouflaged boundaries. Specifically, each block consists of two 1 × 1 convolutions and one 3 × 3 convolution with a residual connection, which enhances local representation capacity while keeping the model efficient. Moreover, the spatial resolution and channel dimension of the CNN features are aligned with those of the Transformer backbone at each stage, so that the subsequent SRA and LHMF modules can perform structurally consistent multi-stage fusion. For the Transformer branch, we adopt the Pyramid Vision Transformer (PVT).

### 3.2 Self-relation attention

To enhance the discriminative capability of feature representations, particularly under complex scenarios such as COD, where foreground and background share highly similar textures, we propose a Self-Relation Attention (SRA) module. This module is designed to selectively emphasize informative spatial responses and suppress irrelevant or noisy activations by modeling the internal relationships within feature maps. The self-relation mechanism computes pairwise affinities between spatial locations, allowing the network to amplify coherent features while suppressing inconsistent responses, crucial for resolving texture ambiguities in camouflage scenarios. The SRA module is lightweight and can be seamlessly integrated into multi-stage feature processing pipelines, making it suitable for both CNN-based local feature branches and Transformer-based global context branches.

**Figure 2:**
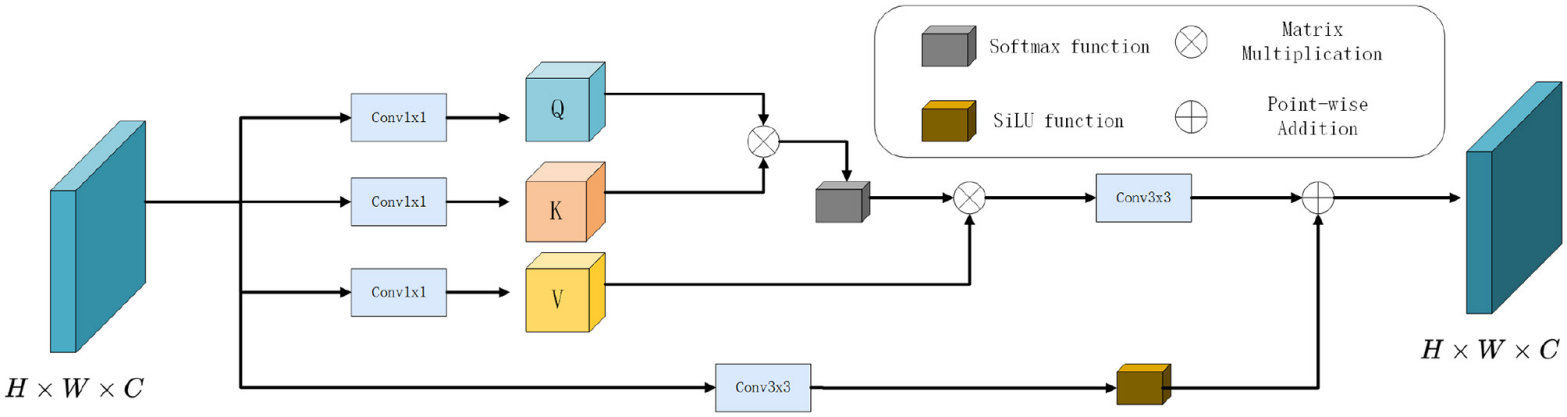
The overall architecture of self-relation attention.

Given an input feature map *F* ∈ *R*^*H*×*W*×*C*^ where *H*, *W*, and *C* denote the spatial height, width, and number of channels, respectively, the SRA module computes a refined representation by capturing intra-feature dependencies via attention mechanisms. The overall architecture is illustrated in Figure 2. The process consists of four key steps: feature projection, attention map computation, relation-guided aggregation, and residual enhancement.

To establish the relational attention, the input feature map *F* is first projected into three distinct embedding spaces using three parallel convolutional layers, yielding the *Q, K, V*. Computed via scaled dot-product attention as shown in Equations 1, 2:


$$\begin{array}{l} Q,K,V=Conv\left(F\right) \end{array}$$ (1)

$$\begin{array}{l} F_{att}=softmax\left(\frac{QK^{T}}{\sqrt{d_{k}}}\right)V \end{array}$$ (2)

To further refine the attended features, *F*_*attn*_ is passed through a convolutional block followed by a non-linear activation. Simultaneously, a parallel residual path is applied directly to the input feature *F*. Both outputs are finally combined via element-wise addition:


$$\begin{array}{l} F_{out}=\sigma \left(Conv\left(F\right)\right)+Conv\left(F_{att}\right) \end{array}$$ (3)

Here, σ denotes the SiLU activation function, defined as


$$\begin{array}{l} \sigma \left(x\right)=x\cdot sigmoid\left(x\right) \end{array}$$ (4)

The use of SiLU improves gradient flow and model expressiveness compared to conventional ReLU or LeakyReLU functions. Its continuous differentiability mitigates sharp saturation effects, facilitating smoother optimization. The residual connection ensures the preservation of original low-level features, while the attention branch brings in semantically enriched context information. This dual-path design balances feature stabilization and contextual refinement, which is vital for handling camouflage-induced feature similarities.

### 3.3 Low-high mix fusion

**Figure 3:**
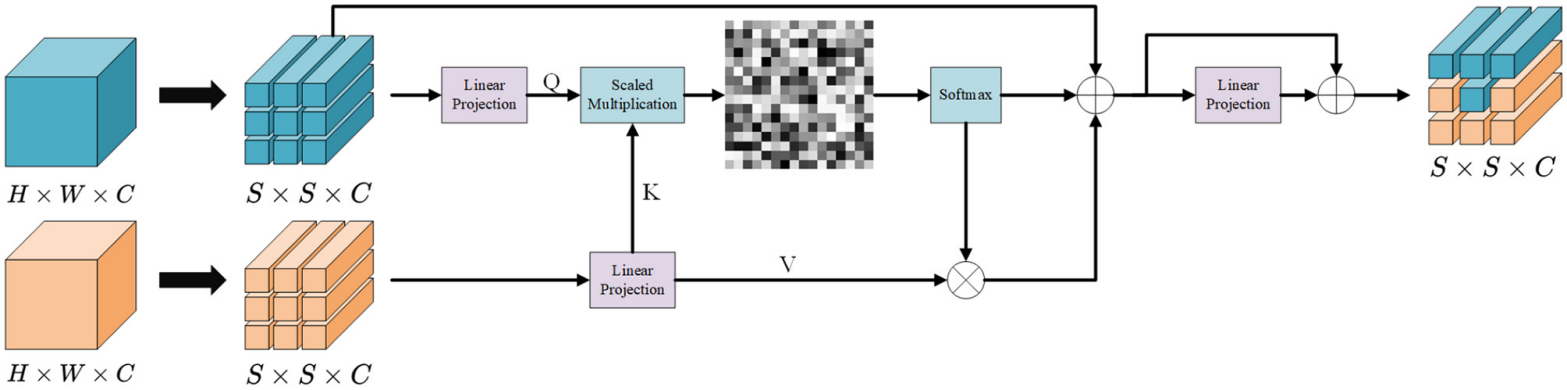
The overall architecture of low-high mix fusion.

To effectively integrate global semantic context and local structural details, we propose the Low-High Mix Fusion (LHMF) module, as illustrated in Figure 3. The LHMF module is designed to fuse the low-frequency global features derived from the original image with the high-frequency local features extracted via the Laplacian transformation. This design enables the network to maintain semantic coherence at the object level while enhancing boundary localization.

Given two feature maps extracted from the same input image, one from the original image and one from its Laplacian-transformed version, the LHMF module performs a cross-attention fusion to capture both coarse contextual representations and fine-grained structural cues. The global features emphasize semantic content and object-level understanding, while the local features focus on boundary sharpness and fine texture, which are often suppressed or missing in conventional CNN backbones when processing camouflage targets. The cross-attention mechanism adaptively weights local features based on global semantic relevance, suppressing boundary noise unrelated to target objects.

Let *F*_*l*_ denote the global feature map extracted from the original image and *F*_*h*_ represent the local feature map derived from the Laplacian-transformed image. Both feature maps are passed through a linear projection and reshaped into sequences for attention computation:


$$\begin{array}{l} F_{l}^{\prime }=Reshape\left(LN\left(F_{l}\right)\right)\in R^{S\times C},F_{h}^{\prime }=Reshape\left(LN\left(F_{h}\right)\right)\in R^{S\times C} \end{array}$$ (5)

The core of the LHMF module lies in the cross-attention mechanism, where the global features serve as the query to attend over the local feature space. Using global features as queries prioritizes boundary integration that aligns with semantic context, avoiding spurious edge distractions. This is formulated as:


$$\begin{array}{l} Q,K,V=Linear\left(F\right) \end{array}$$ (6)

$$\begin{array}{l} F_{fused}=softmax\left(\frac{Q_{l}K_{h}^{T}}{\sqrt{d_{k}}}\right)V_{h} \end{array}$$ (7)

The attention output is then projected back to the original feature dimension and reshaped to the spatial dimension *H* × *W* × *C* before being fused with the global feature through a residual connection:


$$\begin{array}{l} F_{out}=Linearn\left(F_{fused}\right)+F_{fused} \end{array}$$ (8)

The residual connection preserves baseline semantic information while augmenting it with boundary-refined features. This ensures robustness against attention misalignment in noisy camouflage scenarios.

### 3.4 Loss function

In our framework, the loss function is designed to effectively supervise both the COD task and the auxiliary reconstruction task. For the main segmentation objective, we adopt a hybrid supervision strategy commonly used in binary image segmentation tasks such as salient object detection and COD. Specifically, we employ a combination of binary cross-entropy loss and intersection-over-union loss following the practices in previous work. This composite loss, denoted as COD, serves as the principal training objective:


$$\begin{array}{l} L_{COD}=L_{IoU}\left(P^{i},G\right)+L_{BCE}\left(P^{i},G\right) \end{array}$$ (9)

The IoU loss optimizes structural consistency between predictions and ground truth, while BCE ensures pixel-wise discriminability.

To progressively fuse multi-level residual frequency representations during decoding, we impose lateral supervision on the intermediate decoder outputs. Each decoder stage outputs a binary prediction map *P*_*i*_(*i* = 1, 2, 3, 4), with the last one *P*_4_ serving as the final prediction. This strategy ensures that each decoder layer receives effective gradient feedback, promoting better semantic alignment and contour preservation across scales.

In addition to the main segmentation loss, we introduce an auxiliary image reconstruction task to enhance the fusion between residual frequency features and contextual representations learned from separate encoders. For this task, we select three levels of frequency-enhanced features, *R*_1_, *R*_2_, *R*_3_, from the LHMF, as well as the final contextual representation from the transformer-based encoder. These features are passed through a decoder structure to reconstruct the original input image. The decoder uses transposed convolutions and skip connections to upsample features to the input resolution.

Although this regression objective does not directly contribute to the COD task, it enables the model to implicitly learn correspondences between residual frequency and contextual cues. By reconstructing fine-grained image structures, the network gains a stronger understanding of texture and boundary information, both of which are crucial for identifying camouflaged regions. The reconstruction loss is defined using the mean squared error:


$$\begin{array}{l} L_{rec}=\frac{1}{N}\overset{N}{\underset{i=1}{\sum }}||\hat{I_{i}}-I_{i}|{|}^{2} \end{array}$$ (10)

where $\hat{I_{i}}$ denotes the reconstructed image and *I*_*i*_ is the ground truth input image.

Finally, the overall loss function used to train the network is defined as:


$$\begin{array}{l} L_{total}=\overset{4}{\underset{i=1}{\sum }}\frac{1}{2^{i-1}}L_{COD}+\lambda L_{rec} \end{array}$$ (11)

where λ is a balancing weight that controls the contribution of the auxiliary reconstruction loss. This comprehensive supervision strategy effectively enhances the network's ability to localize camouflaged objects while simultaneously enforcing representational consistency across encoders.

## 4 Experiments

### 4.1 Dataset

We conduct comprehensive experiments on three publicly available benchmark datasets: CAMO, COD10K, and NC4K, to validate the effectiveness and generalization ability of our proposed method in diverse camouflage scenarios. The CAMO dataset contains a total of 2,500 images, equally divided into 1250 camouflage images and 1250 non-camouflage images. Among the camouflage images, 1,000 images are used for training, and 250 images are reserved for testing. The COD10K dataset is one of the largest camouflage object detection datasets, comprising 5,066 camouflage images. These are split into 3040 training images and 2026 testing images, covering a wide range of object categories and backgrounds, which increases the diversity and complexity of the detection task. The NC4K dataset contains 4121 camouflage images, which are exclusively used for testing purposes to assess the model's performance in a more challenging and unseen setting. These datasets differ not only in size but also in image characteristics, such as background clutter, object scale, and scene diversity, which makes them ideal benchmarks for evaluating robustness and generalization. In the training phase, we jointly use the 3040 training images from COD10K and the 1,000 camouflage images from CAMO to train our model. This combination allows the model to benefit from the rich variations in object appearance and background context, thereby improving its robustness. During the testing phase, we evaluate the trained model on the respective test sets of CAMO, COD10K, and NC4K. This setup enables us to systematically test both in-domain performance and out-of-distribution generalization capability.

### 4.2 Implementation details

In the training phase, all input images are uniformly resized to 384 × 384. The training is performed with a batch size of 8 using the SGD optimizer, and the initial learning rate is set to 1e-4, with a momentum coefficient of 0.9 and weight decay of 5e-4. To facilitate effective optimization, the learning rate is scheduled to increase linearly during the first 20 epochs, followed by a cosine annealing decay strategy.

### 4.3 Evaluation metrics

To thoroughly evaluate the performance of our model, we adopt four widely recognized quantitative metrics: Structure Measure (*S*_*m*_) Fan et al. (2017): This metric quantifies the structural similarity between the predicted saliency map and the ground truth mask. It focuses on preserving the spatial layout and structural consistency of the predicted camouflage region, especially in terms of object contours and global shape coherence. Adaptive E-measure (α*E*) Fan et al. (2018): This metric combines both global statistics and local pixel-level matching to evaluate the precision and recall in an adaptive manner. By dynamically adjusting the weights based on image content, it provides a more reliable assessment of detection quality across different scenes. Weighted F-measure ($F_{\beta }^{\omega }$) Margolin et al. (2014): Unlike the standard F-measure, the weighted version assigns greater importance to hard-to-detect regions, such as object boundaries or regions with low contrast, making it especially suitable for camouflage object detection tasks. Mean Absolute Error (MAE) Perazzi et al. (2012): This pixel-wise error metric directly measures the average absolute difference between the predicted saliency map and the ground truth, offering a straightforward yet effective way to capture overall prediction bias and quality.

### 4.4 Comparison with state-of-the-arts

We compare a series of state-of-the-art (SOTA) methods, which can be categorized into the following groups: Direct recognition methods, which identify camouflaged objects by directly leveraging semantic information from the input image. Representative methods include FPNet (Song et al., 2023), UJSC (Li et al., 2021), and FSPNet (Huang et al., 2023). Boundary-guided methods, which enhance the recognition of camouflaged objects by employing attention mechanisms to better perceive object boundaries. Examples of this category include BGNet (Sun et al., 2022), EAMNet (Sun et al., 2023), and EANet (Liu et al., 2024). Depth-guided methods, which achieve COD by integrating depth information into the model. Notable approaches include RISNet (Wang et al., 2024) and DaCOD (Wang et al., 2023). Frequency-domain based methods, which utilize various frequency-domain features to boost the detection of camouflaged objects. Representative models include FDNet (Zhong et al., 2022), CamoFA (Le et al., 2025), and FEDER (He et al., 2023). The comparative experimental results are presented in Table 1.

**Table 1:** Comparison of SeCoCR with 19 state-of-the-art models on three COD benchmark datasets for four standard assessment metrics, with the best results highlighted in bold.

**Methods**	**Publications**	**CAMO**				**COD10K**				**NC4K**			
		*S*_*m*_	$E_{\phi }^{a}$	$F_{\beta }^{\omega }$	*M*	*S*_*m*_	$E_{\phi }^{a}$	$F_{\beta }^{\omega }$	*M*	*S*_*m*_	$E_{\phi }^{a}$	$F_{\beta }^{\omega }$	*M*
**Direct detection methods**													
UJSC; (Li et al. 2021)	CVPR2021	0.800	0.853	0.728	0.073	0.809	0.891	0.684	0.035	0.842	0.907	0.771	0.047
PFNet; (Yang et al. 2021)	CVPR2021	0.782	0.855	0.695	0.085	0.800	0.868	0.660	0.040	0.829	0.894	0.745	0.053
SLSR; (Lv et al. 2021)	CVPR2021	0.787	0.855	0.696	0.080	0.804	0.882	0.673	0.037	0.840	0.902	0.766	0.048
PreyNet; (Zhang et al. 2022)	ACM MM2022	0.784	0.859	0.794	0.086	0.817	0.850	0.666	0.036	0.839	0.886	0.746	0.052
SINetV2; (Fan et al. 2021)	TPAMI2022	0.820	0.875	0.743	0.070	0.815	0.863	0.680	0.037	0.847	0.898	0.770	0.048
FPNet; (Song et al. 2023)	CVPR2022	0.844	0.903	0.778	0.062	0.837	0.897	0.731	0.030	0.834	0.895	0.750	0.052
SegMaR; (Jia et al. 2022)	CVPR2022	0.815	0.872	0.742	0.071	0.833	0.895	0.724	0.033	0.841	0.905	0.781	0.046
ZoomNet; (Pang et al. 2022)	CVPR2022	0.820	0.883	0.752	0.066	0.838	0.893	0.729	0.029	0.853	0.907	0.784	0.043
FSPNet; (Huang et al. 2023)	CVPR2023	0.856	0.919	0.799	0.050	0.851	0.900	0.735	0.026	0.879	0.923	0.816	0.035
**Edge-assisted detection methods**													
BSA-Net; (Zhu et al. 2022)	AAAI2022	0.794	0.866	0.717	0.079	0.818	0.894	0.699	0.034	0.841	0.906	0.771	0.048
BGNet; (Sun et al. 2022)	IJCAI2022	0.812	0.876	0.749	0.073	0.831	0.902	0.722	0.033	0.851	0.911	0.788	0.044
MGL; (Zhai et al. 2022)	TIP2022	0.782	0.847	0.695	0.085	0.814	0.865	0.666	0.035	0.833	0.893	0.739	0.053
EAMNet; (Sun et al. 2023)	ICME2023	0.831	0.890	0.763	0.064	0.839	0.907	0.733	0.029	0.862	0.916	0.801	0.040
EANet; (Liu et al. 2024)	ICASSP2024	0.841	0.918	0.793	0.051	0.825	0.910	0.709	0.029	0.860	0.922	0.798	0.039
**Depth-aware detection methods**													
RISNet; (Wang et al. 2024)	CVPR2024	0.870	0.922	**0.827**	0.050	0.873	0.931	**0.799**	0.025	0.882	0.925	0.834	0.037
DaCOD; (Wang et al. 2023)	ACM MM2023	0.855	0.911	0.796	0.051	0.840	0.908	0.729	0.028	0.874	0.923	0.814	0.035
**Frequency-assisted detection methods**													
FDNet; (Zhong et al. 2022)	CVPR2022	0.844	0.903	0.778	0.062	0.837	0.897	0.731	0.030	0.834	0.895	0.750	0.052
FEDER; (He et al. 2023)	CVPR2023	0.807	0.876	0.737	0.069	0.823	0.901	0.715	0.032	0.846	0.912	0.788	0.045
CamoFA; (Le et al. 2025)	WACV2025	0.863	0.927	0.790	0.055	0.864	0.911	0.740	0.025	0.872	0.923	0.801	0.037
Ours	-	**0.871**	**0.929**	0.816	**0.048**	**0.875**	**0.928**	0.773	**0.023**	**0.882**	**0.935**	**0.845**	**0.032**

### 4.5 Ablation studies

#### 4.5.1 Effect of SRA and LHMF

**Figure 4:**
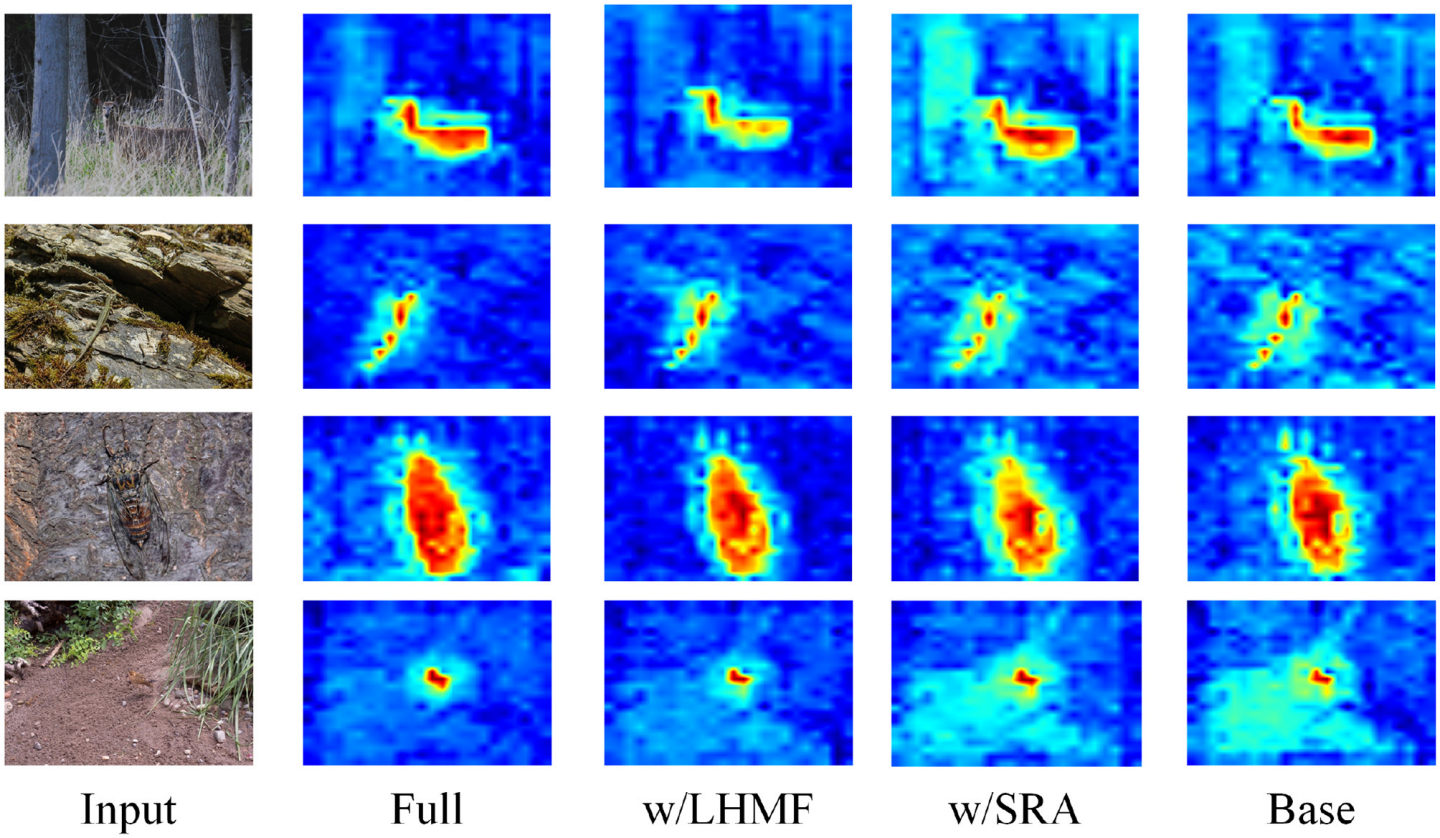
The visualization effect of attention maps in SRA and LHMF ablation studies (w/LHMF, w/SRA denoted with LHMF and with SRA).

Table 2 provides a detailed analysis of the contributions made by the two key modules proposed in our framework: the LHMF module and the SRA module. As shown in the table, the removal of the LHMF module leads to a noticeable drop in performance. This is primarily because the LHMF module introduces Laplacian boundary information, which effectively enhances the representation of object edges and improves the saliency prediction of camouflaged objects. Without this module, the model loses its ability to perceive boundary-aware features, resulting in blurred predictions. Consequently, performance decreases to 0.811, 0.826, and 0.832 on the CAMO, COD10K, and NC4K datasets, respectively. Similarly, when the SRA module is removed, a consistent degradation in performance is also observed. The SRA module is designed to selectively focus on key information embedded within both global and local features, enabling accurate identification of camouflaged targets in complex scenes. Its absence impairs the model's capacity to integrate multi-scale information and differentiate targets from cluttered backgrounds. As a result, the performance drops to 0.841, 0.861, and 0.876 on CAMO, COD10K, and NC4K, respectively. These results clearly demonstrate that both the LHMF and SRA modules play indispensable roles in boosting the overall performance of our framework. The visualization of the ablation studies is shown in Figure 4.

**Table 2:** Effect of SRA and LHMF.

**Base**	**SRA**	**LHMF**	**CAMO**				**COD10K**				**NC4K**			
			*S*_*m*_	$E_{\phi }^{a}$	$F_{\beta }^{\omega }$	*M*	*S*_*m*_	$E_{\phi }^{a}$	$F_{\beta }^{\omega }$	*M*	*S*_*m*_	$E_{\phi }^{a}$	$F_{\beta }^{\omega }$	*M*
✓			0.771	0.834	0.683	0.085	0.814	0.797	0.671	0.071	0.808	0.871	0.723	0.058
✓	✓		0.811	0.885	0.742	0.061	0.826	0.858	0.723	0.060	0.832	0.886	0.803	0.046
✓		✓	0.841	0.904	0.797	0.054	0.861	0.901	0.754	0.035	0.876	0.919	0.833	0.037
✓	✓	✓	**0.871**	**0.929**	**0.816**	**0.048**	**0.875**	**0.928**	**0.773**	**0.023**	**0.882**	**0.935**	**0.845**	**0.032**

The best results are highlighted in bold.

#### 4.5.2 Effect of SRA in each stage

Table 3 presents the performance impact caused by the absence of the SRA module at different stages of the network, thereby verifying the necessity and effectiveness of SRA in each phase. In stage 1, removing the SRA module from the shallow feature extraction layers significantly weakens the model's ability to capture fine-grained local details, such as edges and textures, which are crucial for identifying camouflaged targets. As a result, the performance drops by 0.011, 0.009, and 0.011 on the CAMO, COD10K, and NC4K datasets, respectively. In stage 2, the SRA module is expected to model the relationships between local and global features during mid-level semantic fusion. When it is omitted at this stage, the model struggles to distinguish foreground targets from complex backgrounds, leading to weaker saliency activation and insufficient structural coherence. Under this setting, performance decreases by 0.013, 0.017, and 0.006 across the three datasets. In stage 3, the absence of the SRA module in the deep semantic modeling layers hampers the model's ability to maintain semantic consistency and understand the global contour of objects. This is particularly detrimental in cases involving large-scale or heavily occluded targets. Consequently, the performance further declines by 0.026, 0.019, and 0.013 on CAMO, COD10K, and NC4K, respectively.

**Table 3:** Effect of SRA in each stage.

**Stage1**	**Stage2**	**Stage3**	**CAMO**				**COD10K**				**NC4K**			
			*S*_*m*_	$E_{\phi }^{a}$	$F_{\beta }^{\omega }$	*M*	*S*_*m*_	$E_{\phi }^{a}$	$F_{\beta }^{\omega }$	*M*	*S*_*m*_	$E_{\phi }^{a}$	$F_{\beta }^{\omega }$	*M*
✓	✓		0.845	0.915	0.804	0.053	0.856	0.924	0.766	0.027	0.869	0.933	0.835	0.035
✓		✓	0.858	0.920	0.807	0.053	0.858	0.920	0.768	0.025	0.876	0.932	0.836	0.037
	✓	✓	0.860	0.926	0.806	0.051	0.864	0.923	0.765	0.025	0.873	0.931	0.838	0.034
✓	✓	✓	**0.871**	**0.929**	**0.816**	**0.048**	**0.875**	**0.928**	**0.773**	**0.023**	**0.882**	**0.935**	**0.845**	**0.032**

The best results are highlighted in bold.

### 4.6 Visualization analysis

**Figure 5:**
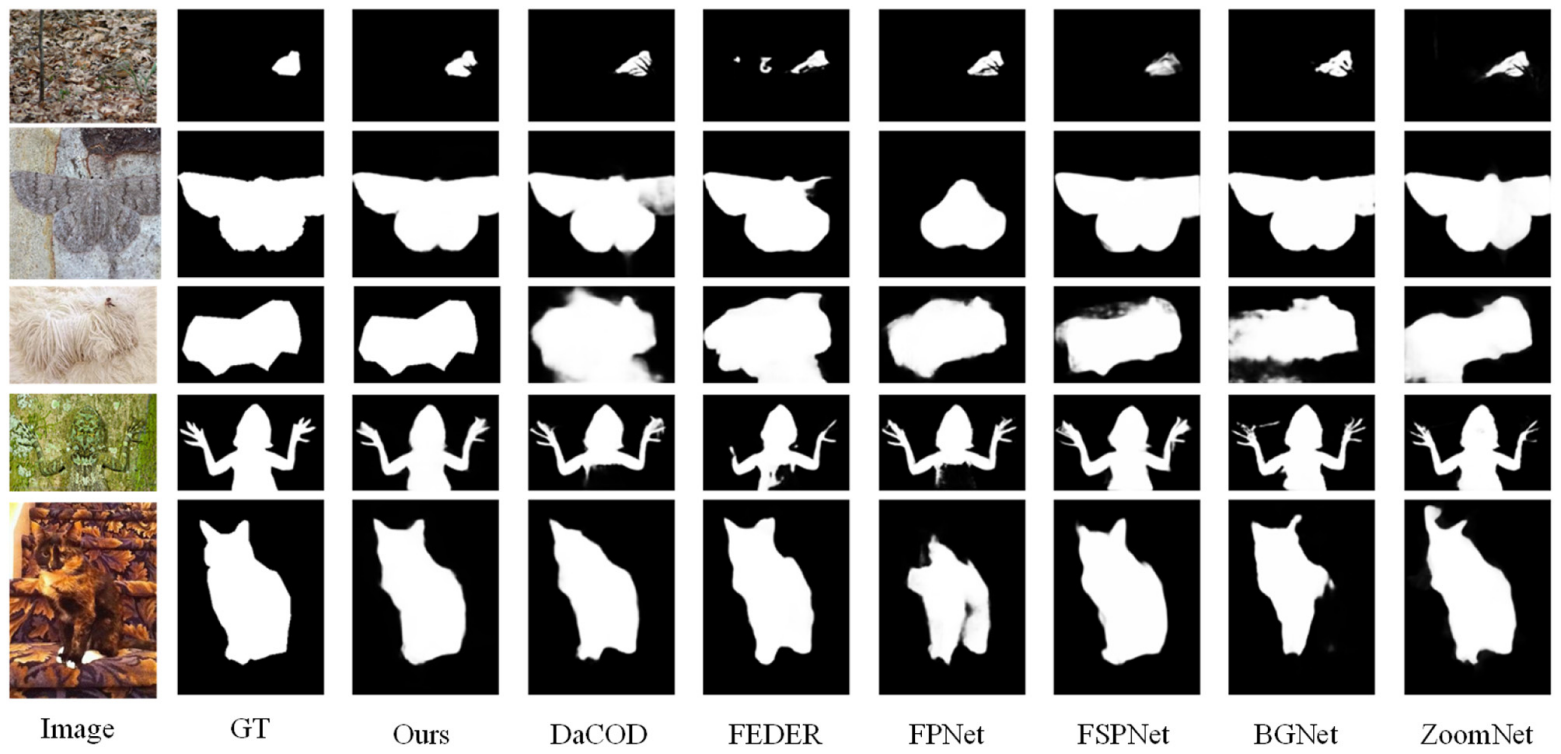
Qualitative comparison between our method and state-of-the-art methods. Compared with depth-aware methods (third column), frequency-based methods (fourth and fifth columns), direct localization methods, and edge-based methods, our method (third column) can segment complete disguised objects in highly disguised scenes.

To visually evaluate the performance of our proposed SeCoCR, we compare it against six representative state-of-the-art methods, including two open-source frequency-domain-based approaches: FDNet and FEDER. The qualitative comparison results are illustrated in Figure 5. As shown in the figure, SeCoCR consistently produces accurate and detailed segmentation results across a wide range of challenging scenarios, including small camouflaged objects, large-scale camouflaged regions, finely textured camouflaged targets, and images containing multiple camouflaged instances. These qualitative results highlight the robustness, accuracy, and superior generalization ability of SeCoCR across diverse camouflage scenarios.

### 4.7 Parameters and FLOPs analysis

As shown in Table 4, our model has higher computational cost and parameter count compared to recent COD architectures. This is due to the increased complexity caused by the additional CNN branches we introduced. This branch explicitly models local texture and fine-grained edge information through the extracted Laplace transform, complementing the global semantic representation of the backbone network, enabling the network to have stronger discriminative ability when dealing with small structures and low contrast areas of disguised targets. Although FLOPs and parameter count are slightly higher than methods such as FEDER, FDNet, FPNet, and FSPNet, the overall scale is still within an acceptable range and will not cause significant inference latency on mainstream GPUs.

**Table 4:** Parameters and FLOPs analysis of our proposed method and multiple COD methods.

	**FEDER**	**FDNet**	**FPNet**	**FSPNet**	**Ours**
Flops(M)	37.37	56.50	47.9	49.6	62.73
Param(G)	23.98	63.22	56.4	58.2	66.99

## 5 Conclusion

This paper proposes a Laplacian-guided dual-branch network that enhances COD by extracting boundary information through Laplacian transformation. The framework incorporates two novel modules: Self-Relation Attention (SRA) and Low-High Mix Fusion (LHM Fusion). The SRA module filters out global and local noise to strengthen the representation of both local and global features. The LHM Fusion module integrates local information from the Laplacian-transformed image with global information from the original image, thereby improving localization and boundary detection accuracy of camouflaged objects. Furthermore, a multi-scale fusion strategy is adopted to strengthen the model's robustness and improve fine-grained prediction accuracy. Extensive quantitative experiments demonstrate the robustness of the proposed method, which significantly outperforms existing frequency-domain-based approaches. In addition, comprehensive ablation studies verify the critical role of each proposed module at various stages of the network.

## 6 Limitations and future work

Although our method achieves superior performance among frequency-domain-based approaches, it still has the following limitations. Due to the introduction of additional modal information and the use of an extra network for feature extraction, our method requires additional computational resources to capture high-frequency boundary information. In future work, we will explore a single-branch, end-to-end approach based on the Laplacian transform to construct a new network for camouflaged object recognition.

## Data Availability

The original contributions presented in the study are included in the article/supplementary material, further inquiries can be directed to the corresponding author.
